# Connections and containers: Using genetic data to understand how watershed evolution and human activities influence cutthroat trout biogeography

**DOI:** 10.1371/journal.pone.0202043

**Published:** 2018-08-10

**Authors:** Kendra R. Eaton, Janet L. Loxterman, Ernest R. Keeley

**Affiliations:** Department of Biological Sciences, Idaho State University, Pocatello, Idaho, United States of America; University of Innsbruck, AUSTRIA

## Abstract

Species with large geographic distributions often exhibit complex patterns of diversity that can be further complicated by human activities. Cutthroat trout (*Oncorhynchus clarkii*) are one of the most widely distributed freshwater fish species in western North America exhibiting substantial phenotypic and genetic variability; however, fish stocking practices have translocated populations outside of their native range and may have obscured intraspecific boundaries. This study focuses on cutthroat trout populations representing three distinct evolutionary clades that are found intermixed within a contact zone between the Bonneville and upper Snake River watersheds in the western United States. We used mitochondrial and microsatellite genetic data, as well as historical stocking records, to evaluate whether populations of cutthroat trout in the contact zone are native or are introduced. We found significant genetic differentiation and fine-scale genetic population structure that was organized primarily by watershed boundaries. While we detected increased genetic diversity in some areas in close proximity to the greatest number of stocking events, the highly organized population structure both within and between areas of the contact zone indicates that the populations are native to the watersheds. Intermixing of distinct evolutionary lineages of cutthroat trout appears to be the result of historical connections between paleodrainages. Our analyses provide a context for understanding how genetic data can be used to assess the status of populations as native or introduced.

## Introduction

Natural geological processes may have a substantial influence on population structure and gene flow by altering the landscape through volcanism, glaciation, mountain building, and plate tectonics [1,2]. Similarly, habitat variability can lead to ecological specialization and genetic differentiation through behavioral, morphological, or physiological adaptation. Given sufficient time, natural isolating mechanisms can lead to local adaptive differentiation and speciation, creating a complex mosaic of unique populations organized by geographic and habitat-related features [2,3]. Although natural processes can sub-divide populations and promote diversification, human activities can obscure natural evolutionary patterns [4–6]. The translocation of species outside of their native range is arguably one of the most important human mediated factors that complicates native species distribution patterns [4,7–9]. For species with extensive geographic structuring, disentangling natural and human-mediated factors affecting their distribution can be difficult, but is critical to the development of management plans for protecting species and their role within ecosystems.

In freshwater ecosystems, natural features commonly isolate populations because many aquatic animals cannot move around physical barriers that extend across the land-water interface [10]. As a result, the contemporary distribution of aquatic taxa is often a reflection of once widely inter-connected populations subsequently isolated by natural events such as major changes in climate and hydrological conditions [11,12]. In western North America, the Great Basin and adjacent regions include a vast area of deserts and mountains with watersheds that have experienced wetter and cooler periods with high levels of connectivity followed by periods of desiccation [13–15]. During pluvial times, lakes covered large areas of the Great Basin allowing widespread dispersal of aquatic species; however, when the climate became more arid, connections were lost and populations isolated [15–17]. Over time, isolated populations accumulated differences as selection acted on adaptive variation or as small populations became subject to genetic drift, creating genetically distinct endemic taxa [2,3]. A variety of aquatic taxa have been identified with localized endemic species in remnant aquatic habitat of arid regions, including amphibians, mollusks, insects, and fish [13,18–20]. The geographic proximity, but phylogenetic distinctiveness of such taxa, often creates a mosaic of adjacent ranges separated by movement barriers across arid landscapes [16,19,20]. Understanding the range and extent of endemic taxa is essential for protecting and conserving native biodiversity. Yet, as human activities continue to expand in areas such as the Great Basin, translocations of closely related species outside of their native range is becoming an increasing concern as it threatens the genetic integrity of native populations, decreasing their abundance through competition or predation [21–24].

In addition to historical geographic features, contemporary processes have been instrumental in shaping the genetic population structure of fish species through various human activities [5,25,26]. Increasingly, the movement of freshwater fish species to areas outside of their native range has become a common occurrence, often to support the demand for recreational fishing opportunities and to supplement natural populations [27]. The cutthroat trout (*Oncorhynchus clarkii*) is one of the most widespread freshwater fish species native to western North America and is also a popular sport fish that has been propagated and translocated from relatively few hatchery stocks [28–30]. Cutthroat trout trace their ancestry in North America to between eight and 16 million years BP [31,32], and as such, natural geological events have influenced their distribution and diversification throughout their range [33]. In western North America, significant changes in watershed connectivity and landscape topology have occurred from processes associated with mountain building, volcanism, and altered flow regimes of rivers during multiple periods of climatic cooling and glaciation [33–38]. As result of these processes, cutthroat trout have diversified into genetically distinct taxa; largely organized by geographic features such as major watershed boundaries [39,40]. Furthermore, the contemporary distribution of cutthroat trout has been complicated by hatchery propagation and translocation to areas outside of their natural range of diversification. While geographic features can largely explain the main axes of cutthroat trout diversification and distribution, overlap in the distribution of some cutthroat trout taxa, and the widespread stocking of hatchery fish have created confusion about whether some cutthroat trout populations are native or have been introduced [6,41,42]. With ongoing efforts to restore and recover endangered cutthroat trout subspecies, determining the extent and frequency of native populations is of vital importance to developing management plans.

**Fig 1:**
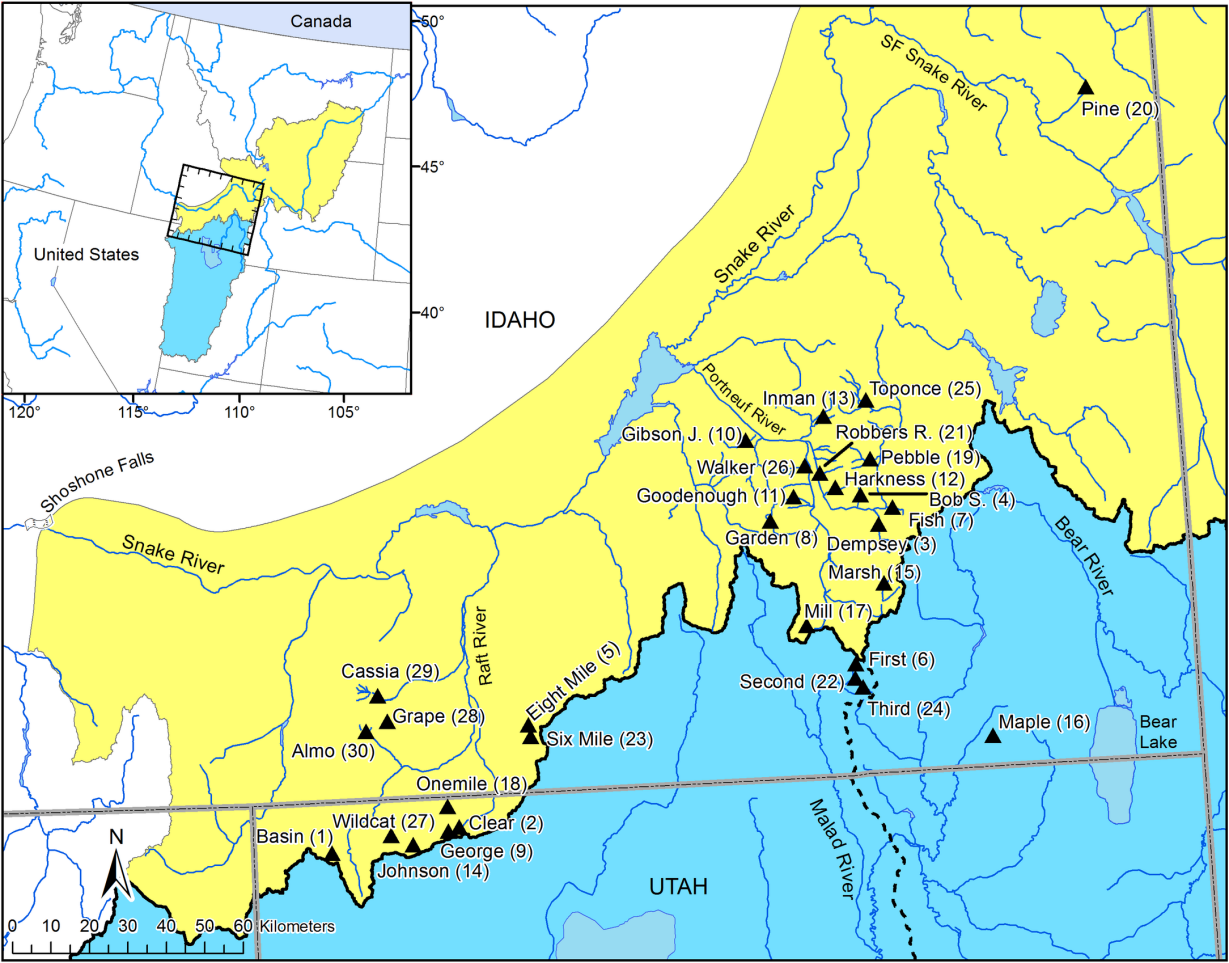
Study area. Sampling locations of cutthroat trout according to stream name within the Bonneville Basin and upper Snake River in western North America. Numbers in parentheses indicate population ID reported in Table 1. Bold line indicates the boundary separating the upper Snake River from the Bonneville Basin. Dashed line represents the division between the Malad River watershed within the Bear River watershed. Inset map indicates estimated boundary for the native range of Bonneville (blue polygon) and Yellowstone (yellow polygon) subspecies of cutthroat trout within the western United States.

Bonneville cutthroat trout (*O*. *c*. *utah*) and Yellowstone cutthroat trout (*O*. *c*. *bouvieri*) are two subspecies whose range is defined by a watershed boundary separating the upper Snake River from the adjacent Bonneville Basin within the Great Basin region of the western U.S. [39,40] (Fig 1). However, even early genetic investigations revealed an additional evolutionary lineage present in the southwestern portions of the Bonneville watershed that is as divergent as populations assigned as Bonneville or Yellowstone cutthroat trout [31,43]. Later genetic studies also documented a distribution of haplotypes thought to be representative of Bonneville cutthroat trout in areas of the upper Snake River [33]. More recent genetic studies of cutthroat trout revealed an intermixing of these evolutionary lineages in a contact zone surrounding the southern portion of the upper Snake River and northern portions of the Bonneville Basin, with one lineage being more closely related to populations from the Colorado River watershed [41]. While similarities of native fish fauna between the Bonneville Basin and upper Snake River have long been associated with pluvial events, such as the Bonneville Flood that connected the two watersheds about 17,400 years ago [44,45], translocations of hatchery trout are common and may also explain unexpected distribution patterns of cutthroat trout subspecies [39,46]. In this study, we used population genetic data to determine if there is evidence for natural admixture of cutthroat trout between the upper Snake River and the adjacent Bonneville Basin or if the intentional translocation of closely related subspecies explains the current distribution of cutthroat trout within the study area.

Genetic analyses provide a powerful tool to resolve the status of populations whose taxonomy and biogeography are poorly understood [47]. Many studies have used genetic approaches to determine source populations of introduced species [48,49], identify invasive species [47,50], or simply to uncover genetic differences that occur between known native and introduced populations [51,52]. In some instances, the distribution of populations is such that it is not clear whether specific populations are native or introduced because individuals may be morphologically indistinguishable from each other despite exhibiting genetic differences [53]. Population genetic data can be used to identify evidence of recent translocations through estimates of genetic differentiation, diversity, and structure [47,48,54,55]. Here, we examine the population genetic structure of cutthroat trout along a contact zone where multiple evolutionary clades are intermixed to determine if secondary contact is a consequence of natural processes or recent human-mediated introductions.

## Materials and methods

### Study area and sample collection

To describe genetic variation of cutthroat trout in the contact zone, we collected tissue samples from individuals representing 30 populations within the Bonneville Basin and upper Snake River (Fig 1). We haphazardly sampled cutthroat trout from headwater streams with backpack electro-fishing. In most instances, we sampled 25 fish from each location, but in four locations we collected fewer fish (Table 1.) Once captured, each fish was fin-clipped for genetic analysis and then released near the point of capture. All sampling locations were presumed to be native populations of cutthroat trout, except Six Mile Creek in the Raft River watershed. Six Mile Creek was chemically treated to remove the fish population because it was introgressed with non-native rainbow trout (*Oncorhynchus mykiss*) and subsequently recolonized using cutthroat trout from neighboring Eight Mile Canyon Creek (D. Megargle, Idaho Department of Fish and Game, Magic Valley Region, personal communication). We included Six Mile Creek as a method for comparison describing the genetic population structure of a known translocated population of cutthroat trout.

**Table 1:** Descriptive measures of genetic variation for each cutthroat trout population and its geographic location.

Population	Watershed	Population ID	n	Mean Ho	Mean Ar	Mean No. of Alleles	No. of mtDNA haplotypes	easting	northing
Basin Creek, ID	Raft	1	25	0.55	4.62	5.27	3	267903	4639520
Clear Creek, UT	Raft	2	25	0.48	3.41	4.00	1	301436	4643950
Dempsey Creek, ID	Portneuf	3	22	0.64	4.31	5.00	2	415889	4714419
E Bob Smith Creek, ID	Portneuf	4	25	0.39	3.31	3.82	1	411772	4722411
Eight Mile Canyon Creek, ID	Raft	5	25	0.25	3.25	4.18	3	321305	4669070
First Creek, ID	Malad	6	25	0.70	5.02	5.73	1	407254	4678708
Fish Creek, ID	Portneuf	7	25	0.54	5.62	6.91	2	419793	4718575
Garden Creek, ID	Portneuf	8	17	0.61	5.10	5.64	2	387831	4717333
George Creek, UT	Raft	9	25	0.66	5.75	7.55	2	298467	4642977
Gibson Jack Creek, ID	Portneuf	10	25	0.61	5.71	7.00	5	383034	4738756
Goodenough Creek, ID	Portneuf	11	25	0.65	5.50	6.91	4	394311	4723259
Harkness Creek, ID	Portneuf	12	22	0.45	3.66	4.18	2	405383	4724780
Inman Creek, ID	Portneuf	13	25	0.68	6.81	8.91	4	403566	4743461
Johnson Creek, UT	Raft	14	25	0.49	4.04	4.64	3	289113	4640137
LHF Marsh Creek, ID	Portneuf	15	25	0.59	4.60	5.64	2	416178	4699105
Maple Creek, ID	Bear	16	25	0.62	5.56	7.09	3	441496	4657545
Mill Creek, ID	Portneuf	17	25	0.67	5.97	7.45	3	395245	4689546
One Mile Creek, UT	Raft	18	25	0.63	4.78	5.55	3	298837	4649457
Pebble Creek, ID	Portneuf	19	25	0.55	5.87	8.00	2	414905	4731530
Pine Creek, ID	SF Snake	20	25	0.67	8.27	11.55	4	478060	4823821
Robbers Roost Creek, ID	Portneuf	21	25	0.56	4.49	5.45	3	401627	4728856
Second Creek, ID	Malad	22	25	0.61	4.13	4.82	2	406940	4675010
Six Mile Creek, ID	Raft	23	25	0.27	2.07	2.27	1	321638	4666072
Third Creek, ID	Malad	24	25	0.54	2.95	3.45	1	408787	4672530
Toponce Creek, ID	Portneuf	25	11	0.62	6.73	6.73	3	414919	4746856
Walker Creek, ID	Portneuf	26	25	0.66	5.38	6.27	2	397871	4730991
Wildcat Creek, UT	Raft	27	25	0.38	1.76	1.82	1	283570	4642956
Grape Creek, ID	Raft	28	25	0.25	3.40	4.18	2	284737	4672669
Cassia Creek, ID	Raft	29	25	0.65	6.75	8.73	5	282795	4679521
Almo Creek, ID	Raft	30	21	0.52	3.99	4.45	2	279037	4670479

n: sample size; Ho: observed heterozygosity; Ar: allelic richness; ID: Idaho; UT: Utah; UTM zone 12

In the laboratory, genomic DNA was extracted from fin clips using the ZR Genomic DNA tissue extraction kit (Zymo Research Corp., Irvine CA) following the manufacturer’s protocol. Fish collection procedures were approved by the Institutional Animal Care and Use Committee at Idaho State University (protocol number 0403509). Fish collection permits were provided under the authority of the Idaho Department of Fish and Game or the Utah Division of Wildlife Resources.

### Mitochondrial DNA data and analyses

We examined the diversity and geographic distribution of cutthroat trout lineages by comparing mitochondrial (mtDNA) haplotypes from all study populations. Mitochondrial DNA was sequenced for 10 individuals from each of the 30 populations (n = 300) for the NADH dehydrogenase subunit 2 gene (ND2). Amplification by polymerase chain reaction (PCR) used the sequencing primers NDintF6 and NDVarR [33,56]. PCR reactions were performed in 25 μl total volumes using 8 μl of 2X ReddyMix PCR Master Mix, 1 μl (10 mM) of each primer, and 2 μl of genomic DNA. The thermal profile included an initial 94°C denaturation step followed by 35 cycles at 94°C for 30 s, annealing at 58°C for 45 s, and extension at 72°C for 75 s, with a final extension at 72°C for 10 min. PCR products were submitted to the Idaho State University Molecular Research Core Facility for purification and DNA sequencing on an ABI 3130*xl* automated sequencer.

Sequences were edited and aligned to a reference cutthroat trout sequence using Sequencer v.4.9 software and Mega v. 6 [57]. We estimated haplotype and nucleotide diversity, as well as haplotype frequency, using DnaSp v. 5.0 [58] and Mega v. 6 software. To illustrate evolutionary relationships, we constructed a phylogenetic tree with representatives of each unique ND2 haplotype from this study, and a single representative sequence from other subspecies of cutthroat trout, including: Colorado River cutthroat trout (*O*. *c*. *pleuriticus*), greenback cutthroat trout (*O*. *c*. *stomias*), Rio Grande cutthroat trout (*O*. *c*. *virginalis*), coastal cutthroat trout (*O*. *c*. *clarkii*), Lahontan (*O*. *c*. *henshawi*), and westslope cutthroat trout (*O*. *c*. *lewisi*), as well as a rainbow trout haplotype used as an outgroup. These supplemental ND2 sequences of cutthroat trout and rainbow trout were obtained from the National Center for Biotechnology Information, GenBank database (http://www.ncbi.nlm.nih.gov/genbank). Phylogenetic trees were constructed using the Tamura-Nei substitution model with invariant sites based on jModeltest [59] results. The final phylogenetic tree was generated with 1000 bootstrap replicates as implemented in the program PhyML [60] and edited in FigTree (ver. 1.4.3; [61]). Mitochondrial DNA sequence diversity was examined at various spatial scales using an Analysis of Molecular Variance (AMOVA) partitioning pairwise differences between sequences among watersheds, among populations within watersheds, and within populations using Arlequin (ver. 3.5; [62]).

### Microsatellite DNA data and analyses

Estimates of genetic population structure, organization, and diversity were compared using nuclear microsatellite loci. All individuals from each population (Table 1) were genotyped for 11 polymorphic loci (Och18, Och24, Och27, Och29, Och30, Och35, Ocl1, Ogo4, Omm1036, Omy77, and Ots107;[63–66]). We amplified microsatellite loci in 15 μl PCR reactions using 6 μl of 2X ReddyMix PCR Master Mix (ABgene), 0.5 μl (10 mM) of each labeled primer, and 2 μl of genomic DNA. A PCR temperature profile for Och18, Och27, Och29, Och30, Och35, Omy77, and Ots107 loci included an initial 94°C denaturation step for 180 s, followed by 40 cycles at 94°C for 30 s, annealing at 53°C for 30 s, and extension at 72°C for 60 s, with a final extension at 72°C for 30 min. To maximize yield of DNA for all remaining loci, we changed the thermal profile to 35 cycles and annealing at 50°C (Ogo4), 57°C (Och24 and Omm1036) and 55°C (Ocl1). All PCR products were submitted to the Idaho State University Molecular Research Core Facility for fragment analysis and genotyping using an ABI3130*xl* automated sequencer. We subsequently used GeneMapper software (ver. 3.7) to genotype every individual at each locus. All peaks were verified manually to ensure accuracy.

Microsatellite diversity and fine scale genetic structure were examined using the number of alleles per loci, average heterozygosity, allelic richness, and pairwise genetic differentiation (F_ST_) with Microsatellite Analyzer (MSA, ver. 4.05; [67]), FSTAT (ver. 2.9.3; [68]), and Arlequin. We used MSA to test for population level differences in the number of alleles per locus and heterozygosity to identify diversity measures that could indicate stocking or natural causes. FSTAT was used to test for significant differences in allelic richness based on 10,000 permutations. Analyses for pairwise genetic differentiation estimates were calculated in Arlequin with 10,000 iterations.

Geographic structuring of genetic data was visualized both at the population level as well as at the watershed level, using a neighbor-joining tree and population assignment tests. If cutthroat trout populations have a natural distribution history, the neighbor-joining tree and clustering should group by watershed and migration should be between neighboring populations. Alternatively, the absence of geographic structure or migration events between watersheds would indicate a significant influence of non-native introductions. For the neighbor-joining tree, we estimated genetic distance using Cavalli-Sforza chord distance [69] and constructed the tree using Phylip (ver. 3.695; [70]). We generated a bootstrap tree using 100 bootstrap replicates and visualized it in FigTree. In addition to illustrating geographic structure using genetic distance, patterns of migration and population clustering were examined using GeneClass2 [71] and Structure (ver. 2.3.4; [72]) software programs. We identified migrants between populations through assignment tests that assign each individual to the most likely population of origin using genetic similarity. To assess geographic genetic structure, we estimated the number of populations (K) with Structure using an individual-based Bayesian assignment method, based on no prior information of population origin. For the Structure analysis, five independent runs for each K (2–30) were conducted using the admixture model at 500,000 iterations with a burn-in of 200,000. The most likely number of population clusters (K) was determined by the estimation of ΔK and the likelihood of the posterior probability [L(K)] [73]. To visualize the assignment of each population in the resulting clusters, we used the programs Clumpp (ver. 1.1.2; [74]) and Distruct (ver. 1.1; [75]).

To examine the degree of geographic structuring and isolation among populations, we compared stream distance and genetic distance between population pairs and tested for associations of genetic data. Stream distance was measured between sampling locations to estimate geographic distance between populations using ArcMap (version 10.3) and the Spatial Tools for the Analysis of River Systems (STARS) extension [76]. Geographic distance between watersheds was calculated by connecting existing rivers to historical linkages through a GIS representation of Lake Bonneville outflow into the upper Snake River. A distance matrix between all sampling locations was obtained using the Spatial Stream Network (SSN) package [77] for R statistical software (ver. 3.3.2). Isolation by distance (IBD) was assessed using 10,000 randomizations with IBD web service [78]. If cutthroat trout populations colonized these areas through watershed connections, we would expect a significant pattern of isolation by distance. Conversely, no relationship between genetic and geographic distance would be expected if the populations were translocated. A Mantel test was used to test for a relationship between genetic distance (F_ST_) and geographic distance (km). To test for associations of genetic data between and within watersheds, we used a principal component analysis (PCA) and an AMOVA to determine if the geographic distribution of microsatellite alleles was primarily organized by watershed boundaries or if they were intermixed across the contact zone. The placement of populations on the principal components axis was based on the similarities across all microsatellite allele sizes. PCA scores were calculated in R statistical software and the average PCA score per population was used to compare population association among locations sampled. For the AMOVA, we examined microsatellite diversity as partitioned among watersheds, among populations within watersheds, and within populations as implemented in Arlequin.

### Stocking data and analyses

If stocking activities have been a primary factor influencing the diversity of trout in the study area, then the frequency and extent of stocking should be related to measures of genetic diversity. To test for an association between stocking history and the population genetic structure of cutthroat trout within the contact zone, we compiled all available historical records of cutthroat trout introductions from the Idaho Department of Fish and Game database (https://idfg.idaho.gov/fish/stocking). Stocking records from the Snake, Southeast, and Magic Valley regions were compiled by waterbody name for all years available in the database (1967–2016) and used to assign the site of translocation within upper Snake River and northern Bonneville watersheds. While most of the study area is covered by the Idaho database, a small portion of the Snake River watershed occurs in the northern extent of Utah (Fig 1). For these populations there are no electronic records available; however, past reviews of the area indicate no stocking of cutthroat trout [79]. We used a geospatial database to visualize the distribution, frequency, and distance of stocking events to the sampling locations. Each location was standardized to the smallest scale watershed boundary dataset layer (12-digit hydrological unit code, HUC) available from the US Watershed Boundary Dataset [80]. We estimated the total number of cutthroat trout stocked at a location by summing the number of fish listed for each stream, river, lake, or reservoir site. We also estimated the frequency of events by counting the number of times stocking occurred at a location for the 49 years of data available at the time we compiled the records. To test for any association between stocking history and genetic diversity, we compared three measures of genetic diversity for each population (allelic richness, number of alleles, and heterozygosity) with the three measures of stocking extent and intensity (distance to nearest stocking location, total number of fish, and the number of events). All three stocking variables were also compared with mitochondrial measures consisting of the number of haplotypes detected in a population. We used a simple and multiple regression analysis to test for the effect of each variable on genetic diversity of cutthroat trout. Tests of significance for each stocking variable were based on a type III sum of squares, implemented in R statistical software.

## Results

Over all cutthroat trout populations sampled for this study, 14 locations were in the Portneuf River watershed, 11 were in the Raft River watershed, and three were in the Malad River watershed (Table 1, Fig 1). Two additional populations outside the contact zone were also included: one tributary to the South Fork of the Snake River (Pine Creek), and one tributary to the Bear River (Maple Creek) of the Bonneville Basin.

### Mitochondrial DNA

Mitochondrial sequences were generated at the ND2 gene (1056bp) for 300 cutthroat trout representing 30 populations, and yielded 18 different haplotypes (Table 2). The number of haplotypes detected per population ranged from one to five and were differentially distributed among populations (Table 1). The most frequent haplotype (H8) occurred in 57 individuals from 12 different populations, while the least frequent haplotypes (H7, H12, H17, and H18) were found in a single individual (Table 2).

**Table 2:** Eighteen identified ND2 haplotypes and their frequency among 30 populations of cutthroat trout within the Bonneville Basin and upper Snake River.

Population	Haplotypes																		
	H1	H2	H3	H4	H5	H6	H7	H8	H9	H10	H11	H12	H13	H14	H15	H16	H17	H18	Lineages
Basin Creek	0	0	0	0	1	0	0	7	0	0	0	0	2	0	0	0	0	0	B, GB
Clear Creek	10	0	0	0	0	0	0	0	0	0	0	0	0	0	0	0	0	0	B
Dempsey Creek	0	0	0	0	0	0	0	2	8	0	0	0	0	0	0	0	0	0	GB
E. Bob Smith Creek	0	0	0	0	0	0	0	10	0	0	0	0	0	0	0	0	0	0	GB
Eight Mile Can.Creek	2	0	0	0	4	0	0	4	0	0	0	0	0	0	0	0	0	0	B, GB
First Creek	0	0	10	0	0	0	0	0	0	0	0	0	0	0	0	0	0	0	GB
Fish Creek	0	0	0	0	0	0	0	4	6	0	0	0	0	0	0	0	0	0	GB
Garden Creek	0	8	0	0	2	0	0	0	0	0	0	0	0	0	0	0	0	0	B
George Creek	0	0	0	0	7	0	0	0	0	0	0	0	0	0	3	0	0	0	A, B
Gibson Jack Creek	0	0	0	0	0	1	0	0	1	0	6	0	0	0	0	0	1	1	A, B, GB
Goodenough Creek	0	0	0	0	1	0	0	1	2	0	6	0	0	0	0	0	0	0	A, B, GB
Harkness Creek	0	0	0	0	0	0	0	8	2	0	0	0	0	0	0	0	0	0	GB
Inman Creek	2	0	0	0	3	0	0	4	0	1	0	0	0	0	0	0	0	0	B, GB
Johnson Creek	1	0	1	0	0	0	0	0	0	0	0	0	8	0	0	0	0	0	B, GB
LHF Marsh Creek	0	0	4	0	6	0	0	0	0	0	0	0	0	0	0	0	0	0	B, GB
Maple Creek	8	2	0	0	0	0	0	0	0	0	0	0	0	0	0	0	0	0	B
Mill Creek	0	0	1	0	2	0	0	7	0	0	0	0	0	0	0	0	0	0	B, GB
One Mile Creek	6	0	1	0	0	0	0	0	0	0	0	0	0	3	0	0	0	0	B, GB
Pebble Creek	0	0	0	0	0	0	0	6	4	0	0	0	0	0	0	0	0	0	GB
Pine Creek	0	0	0	5	3	1	1	0	0	0	0	0	0	0	0	0	0	0	A, B, GB
Robbers Roost Creek	6	0	0	0	3	0	0	1	0	0	0	0	0	0	0	0	0	0	B, GB
Second Creek	0	0	6	0	0	0	0	0	0	0	0	0	0	0	0	4	0	0	GB
Six Mile Creek	10	0	0	0	0	0	0	0	0	0	0	0	0	0	0	0	0	0	B
Third Creek	0	0	10	0	0	0	0	0	0	0	0	0	0	0	0	0	0	0	GB
Toponce Creek	0	0	0	0	0	0	0	3	5	0	2	0	0	0	0	0	0	0	A, GB
Walker Creek	0	0	0	0	2	0	0	0	0	8	0	0	0	0	0	0	0	0	B
Wildcat Creek	0	0	0	0	0	0	0	0	0	0	0	0	10	0	0	0	0	0	GB
Grape Creek	2	0	0	0	0	0	0	0	0	0	0	0	8	0	0	0	0	0	B, GB
Cassia Creek	1	0	1	0	5	2	0	0	0	0	0	1	0	0	0	0	0	0	A, B, GB
Almo Creek	1	0	9	0	0	0	0	0	0	0	0	0	0	0	0	0	0	0	B, GB
Total	49	10	43	5	39	4	1	57	28	9	14	1	28	3	3	4	1	1	

GB: Great Basin lineage; A: Bonneville-Yellowstone lineage, clade A; B: Bonneville-Yellowstone lineage, clade B

**Fig 2:**
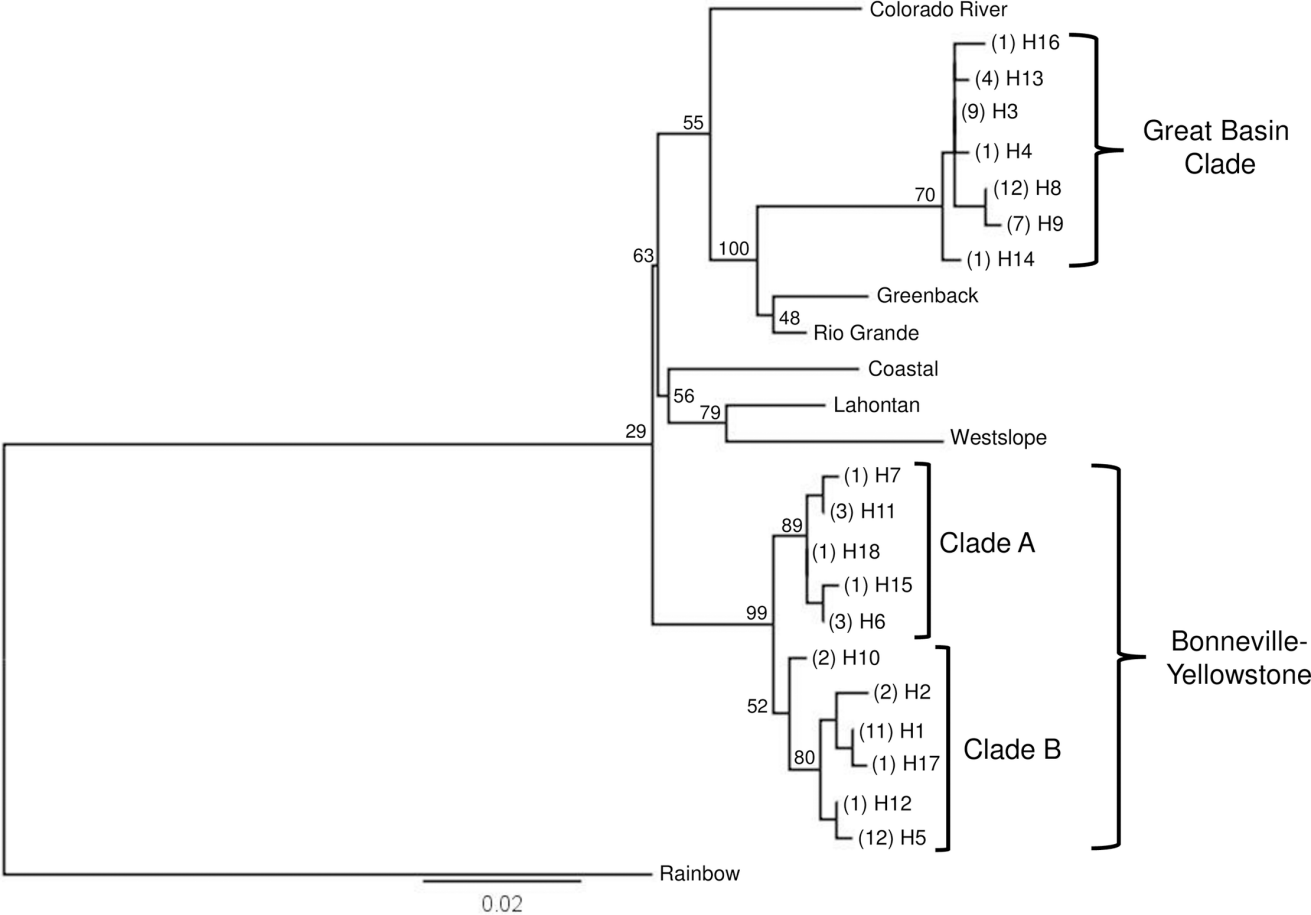
Cutthroat trout phylogeny. Maximum likelihood phylogeny of cutthroat trout within the Bonneville Basin and upper Snake River based on the mitochondrial ND2 gene in reference to other subspecies of cutthroat trout obtained from Genbank. Numbers in parentheses represent the number of sampling locations where the haplotype was detected followed by the haplotype number. Numbers on branches indicate percent bootstrap support based on 1000 replicates. Scale bar represents the proportion of sequence divergence in the ND2 gene used to construct the phylogeny.

Phylogenetic comparisons using the 18 different haplotypes identified in this study and including representative samples from other cutthroat trout subspecies as well as rainbow trout, revealed two distinct clades within the contact zone. (Fig 2). This branching pattern recovers the same division detected in previous analyses by Loxterman and Keeley [41] and is referred to as the Great Basin clade and Bonneville-Yellowstone clade. In this study, as in the past analysis, the Bonneville-Yellowstone clade is further subdivided into two well-supported subclades based on 11 different haplotypes. The second primary or Great Basin clade includes seven different haplotypes and shares a closer evolutionary relationship with subspecies of cutthroat trout from the Colorado River and adjacent watersheds (Fig 2). Of the 18 different haplotypes we detected in the contact zone, 15 haplotypes were identical to sequences previously deposited in Genbank; however, three new haplotypes were identified from One Mile Creek (H14), Second Creek (H16), and Pine Creek (H7; Table 2).

**Fig 3:**
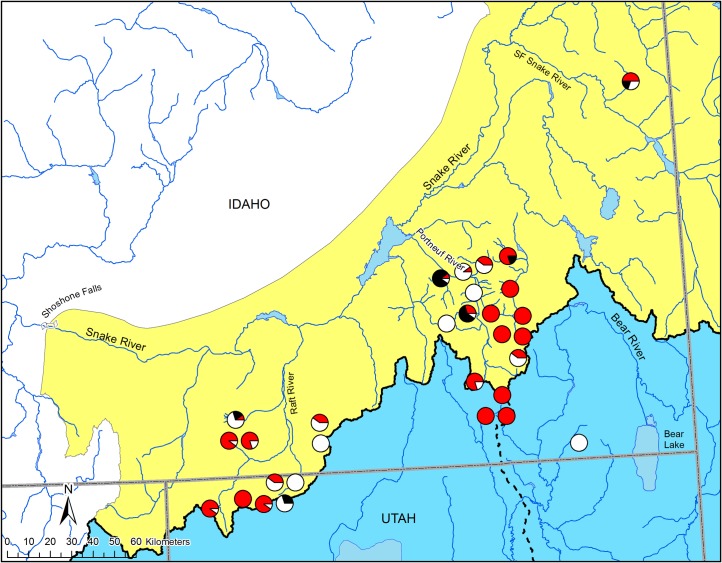
Mitochondrial DNA haplotype distributions. Geographic distribution of the two major lineages of Bonneville-Yellowstone and Great Basin lineages of cutthroat trout (see Fig 2) depicted by the red (Great Basin clade), black (Bonneville-Yellowstone clade A), and white (Bonneville-Yellowstone clade B) proportions of each circle. Colored polygons represent the estimated boundary for the native range of Bonneville (blue) and Yellowstone (yellow) subspecies of cutthroat trout. Dashed line represents the division between the Bear River and Malad River watersheds.

Geographic structuring of haplotype distribution in the contact zone was evident within and among watersheds. All populations from the Malad River watershed were associated with the Great Basin clade (Fig 3). While most of the populations from the eastern Portneuf River identified as Great Basin, the western Portneuf River populations grouped in the Bonneville-Yellowstone clade (Fig 3). Similarly, populations from the Raft River watershed grouped with both the Great Basin and the Bonneville-Yellowstone clades; however, populations in the eastern Raft River watershed from more downstream locations associated predominantly with the Bonneville-Yellowstone clade, while western populations from more upstream locations identified with the Great Basin clade (Fig 3). Based on an AMOVA, differences among watersheds accounted for 8% of the variation in the ND2 sequence diversity (Table 3A). Variation among populations within watersheds accounted for 56% of the diversity, while within population variation explained 36% of the sequence diversity (Table 3A).

**Table 3:** Analysis of molecular variance (AMOVA) among populations of cutthroat trout from three watersheds. Sources of genetic variation were estimated among watersheds, among populations within watersheds, and within populations based on (a) mtDNA pairwise sequence diversity and (b) nuclear microsatellite allele frequencies.

Source of Variation	df	Sum of squares	Percent variation	P-value
(a)				
Among watersheds	2	208.081	8.14	0.12
Among populations within watersheds	25	1228.26	55.97	< 0.001
Within populations	252	746.00	35.89	< 0.001
Total	279	2282.34		
(b)				
Among watersheds	2	564.92	12.14	<0.001
Among populations within watersheds	25	1273.35	20.90	< 0.001
Within populations	1308	4204.75	66.96	< 0.001
Total	1335	6043.012		

### Microsatellite DNA

A total of 718 cutthroat trout were genotyped from the contact zone at 11 microsatellite loci. In most cases 25 individuals were genotyped from each stream, but in five locations we had fewer fish available (range: 11–25, Table 1). All loci were polymorphic, with the average number of alleles per locus ranging from 1.82 in Wildcat Creek to 11.55 in Pine Creek. Allelic richness was also lowest in Wildcat Creek (1.76) and highest in Pine Creek (8.27). Overall, heterozygosity ranged from 0.25 in Grape Creek to 0.70 in First Creek (Table 1). Estimates of pairwise genetic differentiation (F_ST_) indicate significant differentiation between all 30 population pairs. The lowest genetic differentiation occurred between neighboring populations, Fish Creek and Pebble Creek (F_ST_ = 0.045) and was highest between two Raft River populations, Six Mile Creek and Wildcat Creek (F_ST_ = 0.67). Across all populations, average F_ST_ was 0.28 (Sl Table 1).

**Fig 4:**
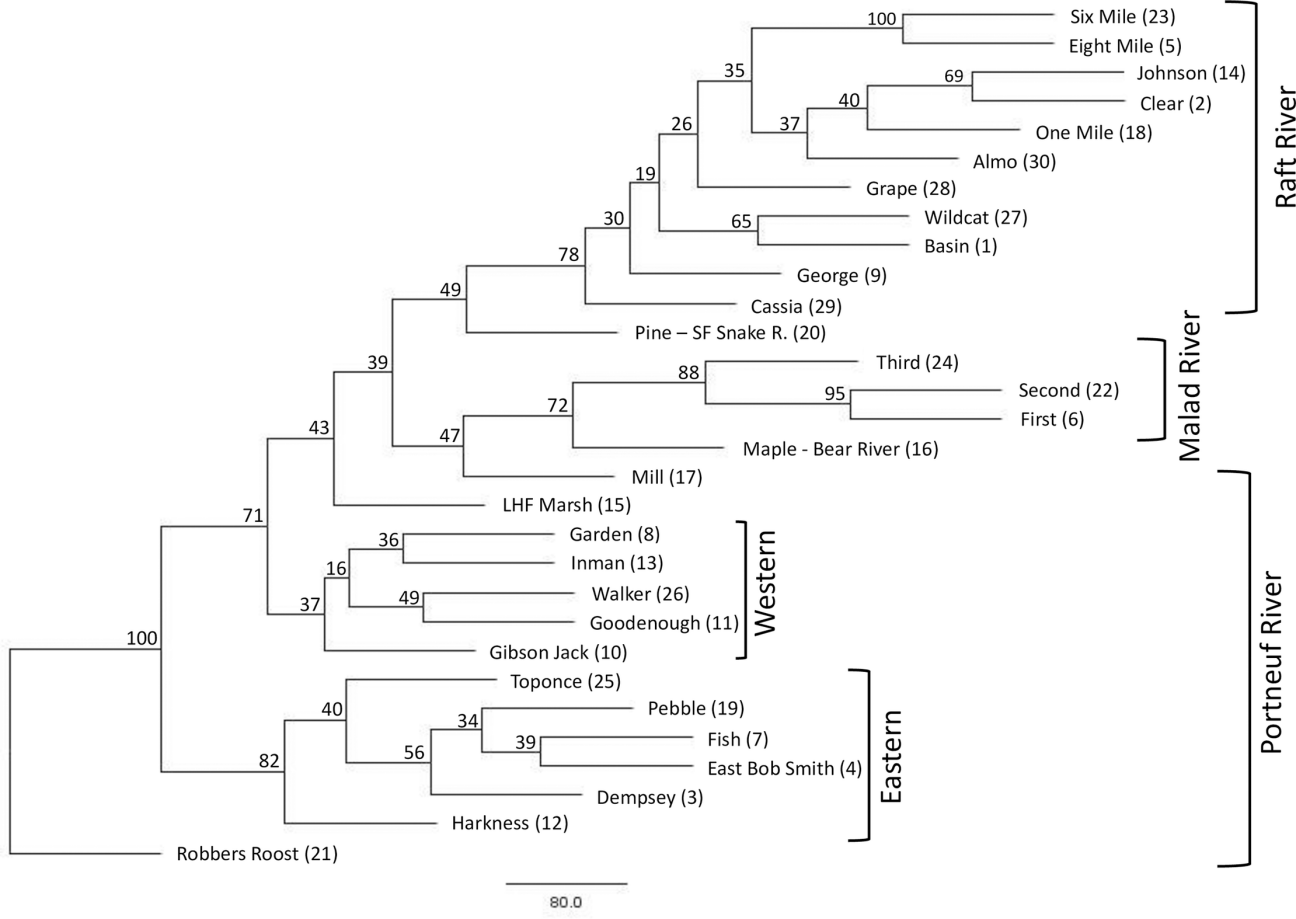
Organization of cutthroat trout populations. Neighbor joining tree of the 30 populations (population ID) of cutthroat trout sampled from the Bonneville Basin and upper Snake River based on Cavalli-Sforza chord distances. Groupings of major watersheds are displayed in brackets. Numbers represent the percentage of supported bootstraps.

Despite that many of the nodes in the neighbor-joining tree exhibit weak bootstrap support, the general trends reflect the geographical distribution of the populations sampled. In the tree, the primary divergence among populations separated most of the upper Portneuf River on the east side of the valley from all other populations (Figs 3 and 4). One Portneuf population, Robbers Roost, did not cluster with any other population. Secondary divergence in the tree separated downstream and western Portneuf River populations from those in the Malad River of the Bonneville Basin, as well as the two upper Marsh Creek populations of the Portneuf Valley. All Raft River populations clustered together in the tree and tended to be organized primarily by geography. Cassia Creek, the most downstream population, was most divergent from the others in the Raft River, while the remaining populations were closely associated with neighboring populations in the watershed (Fig 4).

**Fig 5:**
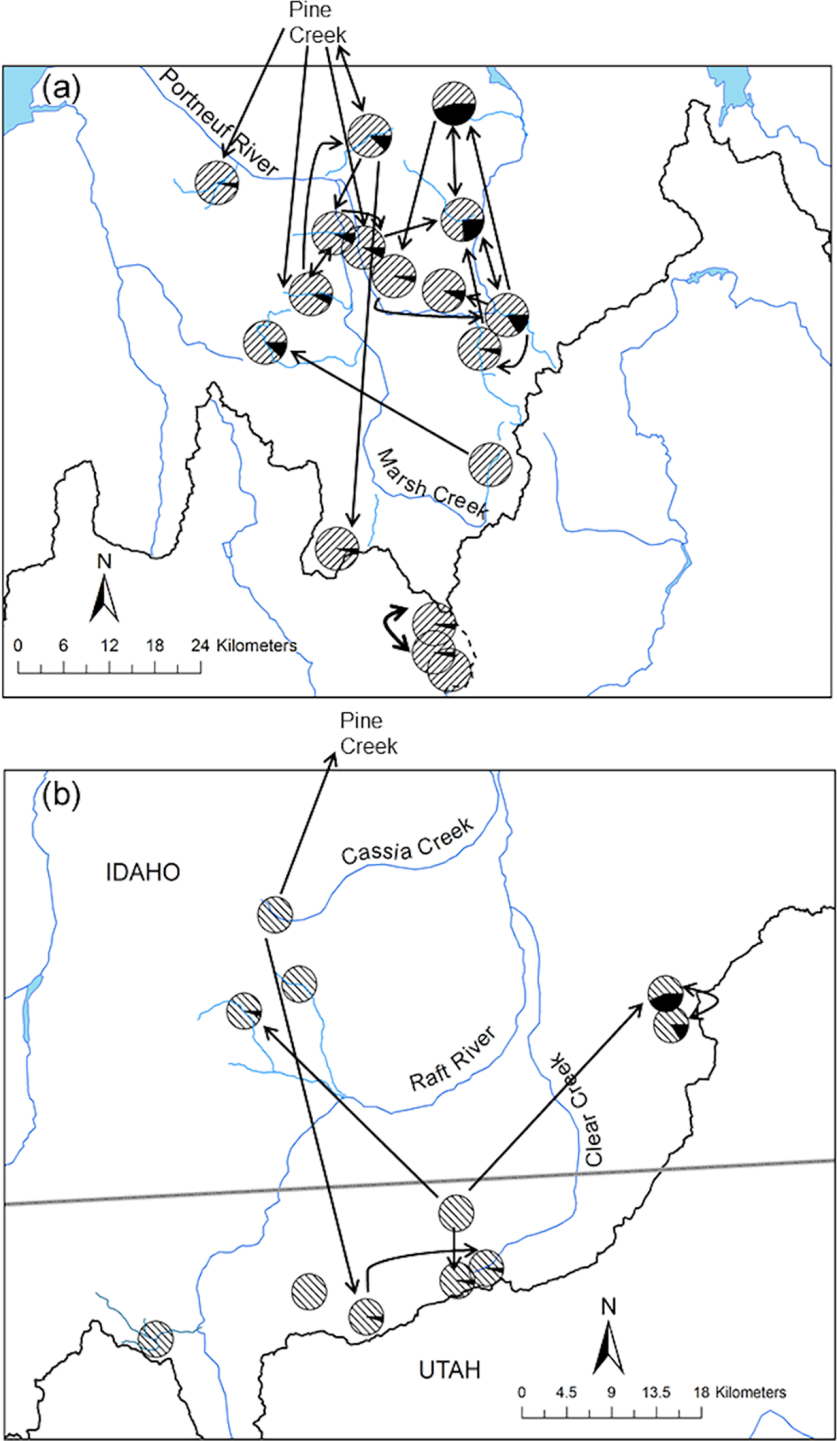
Proportion of assignments by location. Location and assignments of cutthroat trout within the Bonneville Basin and upper Snake River based on 11 microsatellite loci. Hatching represents the proportion of individuals that assigned to their sampled population. Solid black represents the proportion of individuals that assigned to a different population than what was sampled. Arrow direction points from the location where a misassigned individual originated to their sampled location within the **(a)** Portneuf and Malad River watersheds and **(b)** the Raft River watershed.

On average, 92% of individuals assigned to their sampling location (range 55–100%). While some migration was detected, most of these events occurred between neighboring populations (Fig 5). Not surprisingly, the largest number of misassignments was detected between adjacent populations in the Portneuf River. Most of these streams are perennially or seasonally flow-connected or would have been within the last 50–100 years. No recent migration was detected between the Portneuf River and Malad River or Raft River populations or between the Raft River and Malad River populations (Fig 5A and 5B). Very little migration was detected in the Raft River populations and five locations had no misassignments (Fig 5B). Most migrants in the Raft River were between Eight Mile Canyon Creek and neighboring Six Mile Creek (Fig 5B).

**Fig 6:**
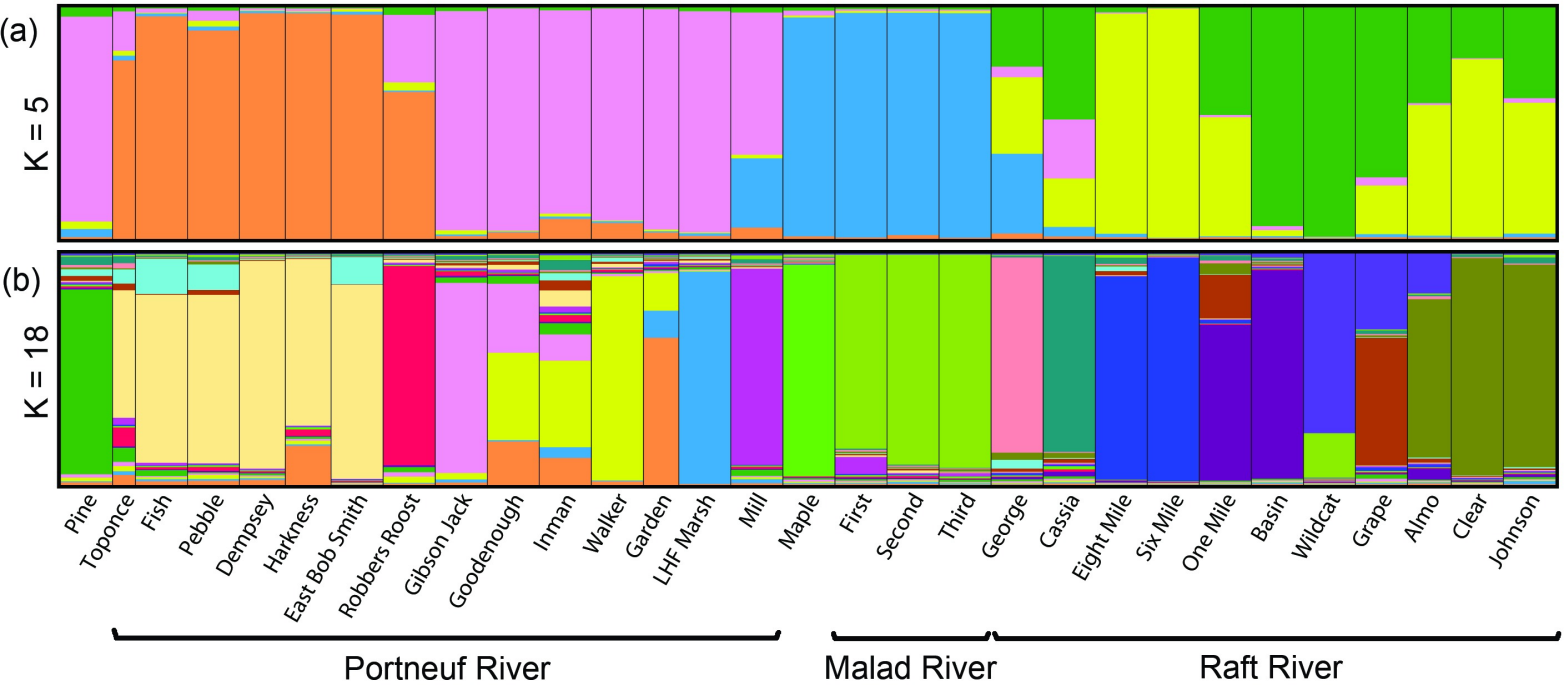
Structure assignments. Proportions of assigned clusters for populations of cutthroat trout within the Bonneville Basin and upper Snake River based on 11 microsatellite loci. Clusters and proportions were calculated in Structure with **(a)** K = 5 and **(b)** K = 18. Each color represents a different cluster. Groupings of watersheds are displayed within brackets.

Bayesian cluster analyses of the 30 trout populations suggested the most likely number of clusters, based on the data, was K = 5 or K = 18 (Fig 6). Results based on five clusters divided the 30 populations largely based on geographic location within the watersheds. The clusters included eastern Portneuf River, western Portneuf River, Malad River, and two groupings within the Raft River. In all cases, intermixing primarily occurred within watersheds (Fig 6A). On average, 89.6% of individuals (range: 62.0–99.0%) were assigned to groups of populations from the same watershed. At K = 18, greater levels of intermixing occurred; however, the pattern of geographic structure observed for this level of organization were similar to those for K = 5. The dominant proportion of individuals (mean: 81.2%, range: 47–97%) were assigned to a cluster for a single population or for a group of populations from neighboring locations within a watershed (Fig 6B).

**Fig 7:**
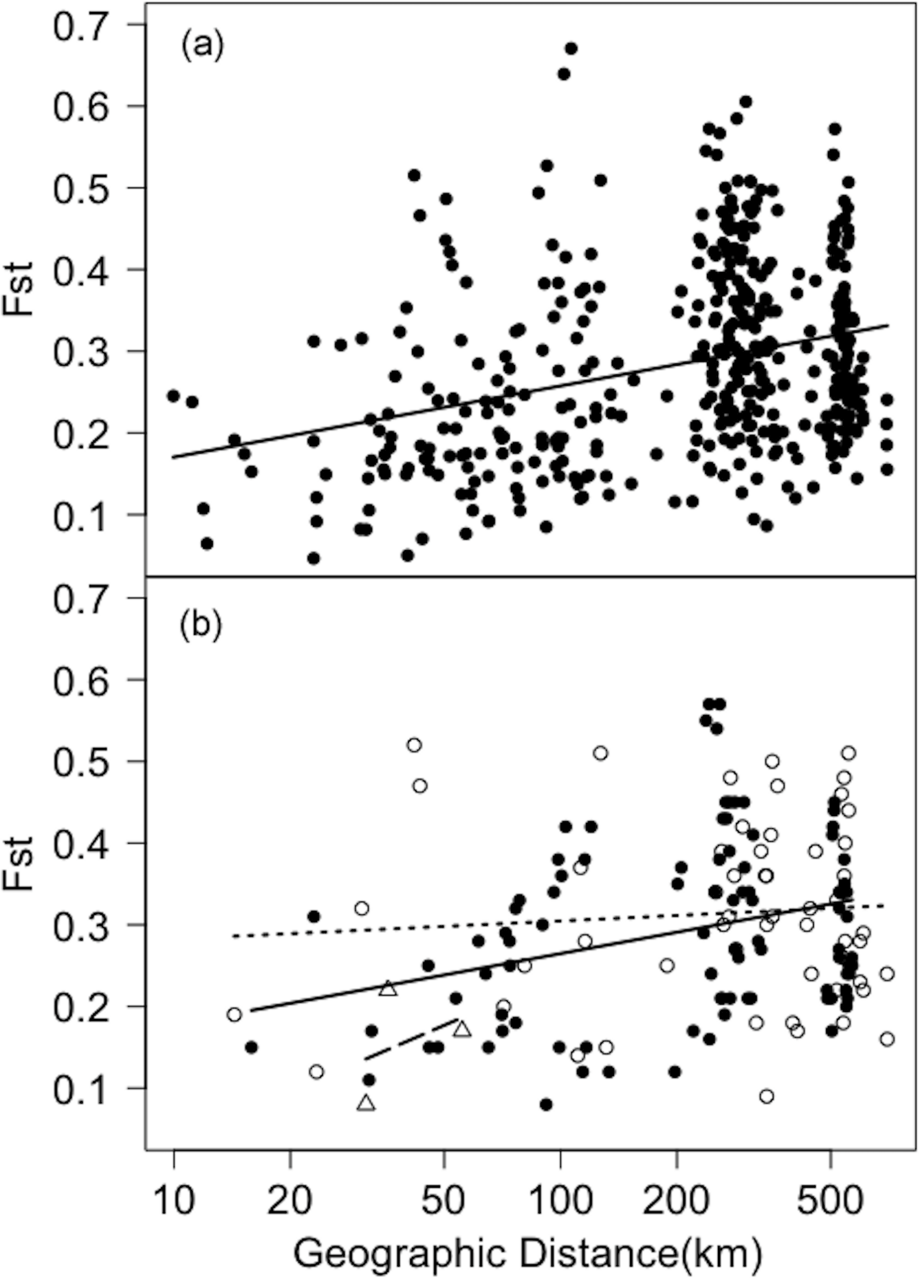
Geographic distance versus genetic difference. (a) The relationship between geographic distance (km) and genetic distance (F_ST_) based on populations of cutthroat trout within the Bonneville Basin and upper Snake River. (Mantel test, r = 0.31, p = 0.0020). (b) The relationship between geographic distance (km) and genetic distance (F_ST_) based on populations of cutthroat trout in each watershed within the Bonneville Basin and upper Snake River. Solid circles and solid line represent the Portneuf River watershed (Mantel test, r = 0.30, p = 0.022), open circles and dotted line represent the Raft River watershed (Mantel test, r = 0.085, p = 0.26, and open triangles and dashed line represent the Malad River watershed (Mantel test, r = 0.37, p = 0.52).

**Fig 8:**
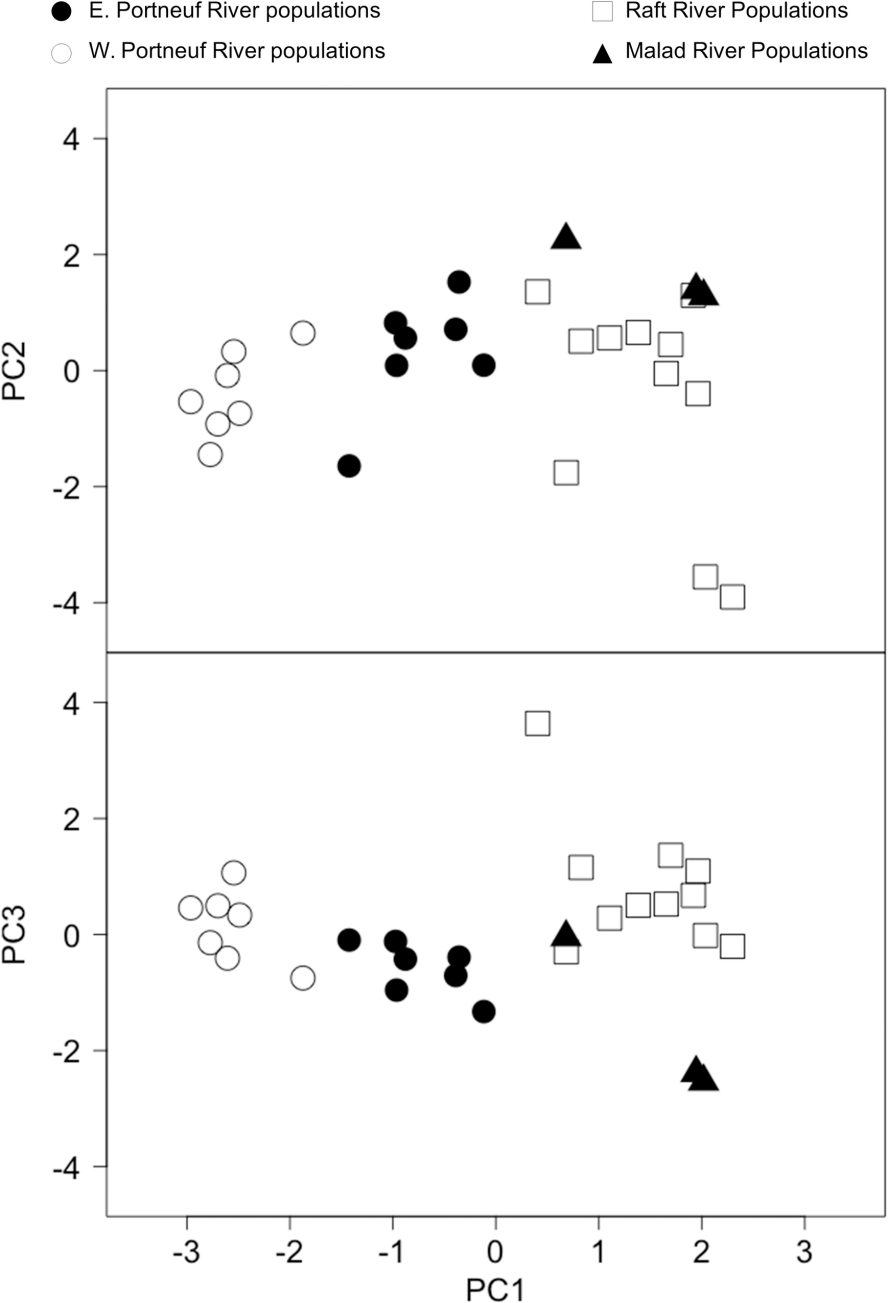
Principal component analysis of microsatellite loci. Principal component analysis for cutthroat trout within the Bonneville Basin and upper Snake River based on 11 microsatellite loci. Populations grouped by major watersheds represented by different clusters. Open circles represent the eastern Portneuf River watershed populations, closed circles represent the western Portneuf River watershed populations, open squares represent the Raft River watershed, and closed triangles represent the Malad River watershed.

The degree of geographic structuring and isolation among populations is further supported by comparisons of geographic distances and genetic distance between population pairs. Across all populations sampled, isolation by distance tests reveal that a significant proportion of the genetic variation between populations is explained by geographic distance (Mantel test, r = 0.31, p = 0.0016; Fig 7A). Within watersheds, geographic distance explains a significant proportion of the variation in genetic distance in the Portneuf watershed (Mantel test, r = 0.30, p = 0.022). We did not detect a significant correlation between geographic distance and genetic distance in the Raft River (Mantel test, r = 0.085, p = 0.26) or Malad River populations (Mantel test, r = 0.37, p = 0.52; Fig 7B). Principal Component Analysis also revealed significant population structuring both within and between the three major watersheds. The first four axes of the PCA explained 47.2% of the genetic variation: PC axis 1 (16.7%), PC axis 2 (12.8%), PC axis 3 (9.46%), and PC axis 4 (8.28%). The first axis separated the Portneuf River from the Raft River and Malad River watersheds, as well as the eastern and western populations of the Portneuf River watershed (Fig 8). The second (Fig 8A) and third axes (Fig 8B) further organized populations within watersheds and separated Raft River populations from Malad River populations. Over the contact zone, 12% of genetic variation occurs among watersheds, 21% of genetic variation occurs among population within watersheds, while 67% of genetic variation exists within individual populations as indicated by an AMOVA (Table 3B).

### Stocking records

**Fig 9:**
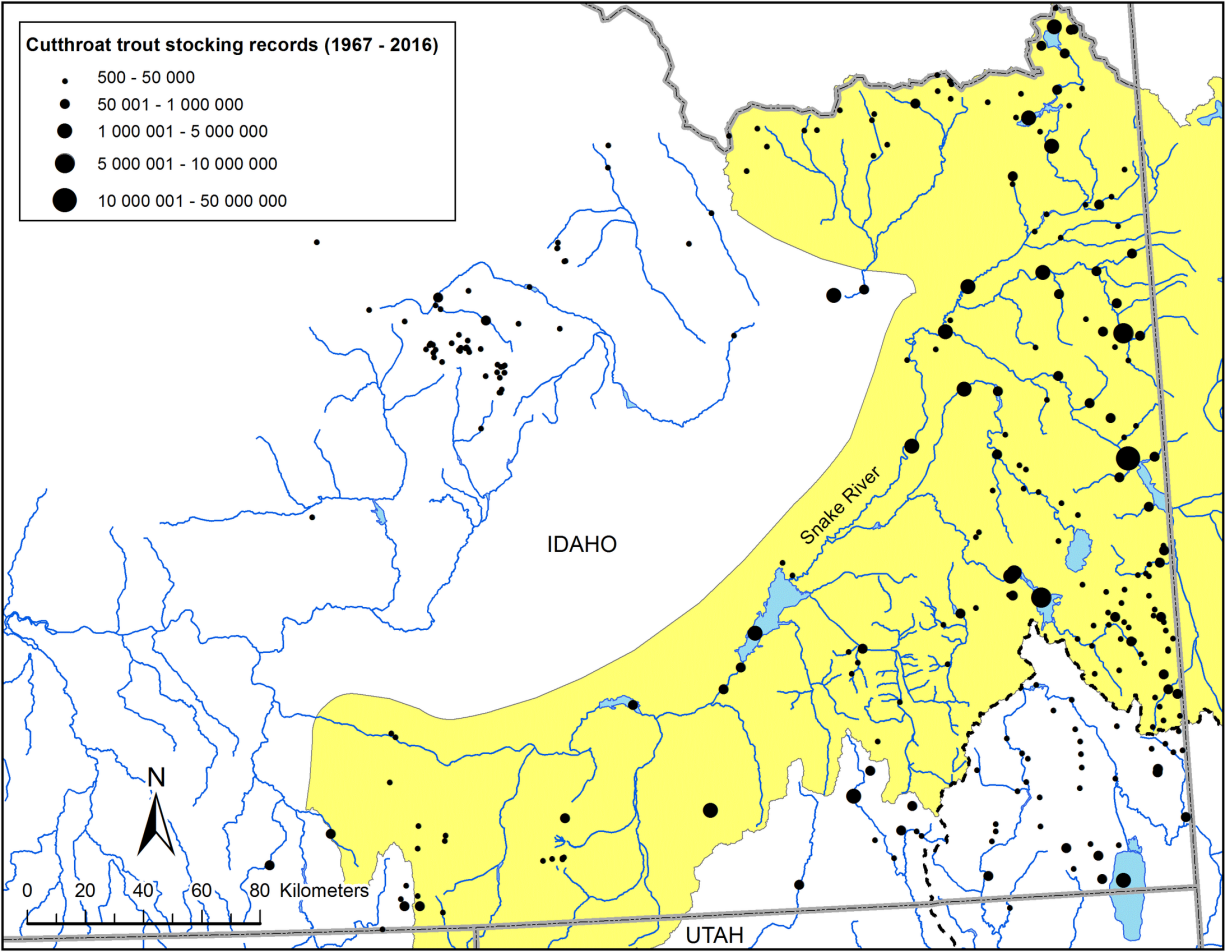
Geographic distribution of historical stocking data for cutthroat trout. Total number of cutthroat trout stocked into the upper Snake, southeast Idaho, and Magic Valley watersheds according to locations reported in the Idaho Department of Fish and Game historical stocking records database from the years 1967 to 2016. See legend for estimated range of fish numbers stocked within each site. Dashed line represents the Bear River watershed boundary.

Based on stocking records available from the Idaho Department of Fish and Game for the years 1967 to 2016, about 123 million cutthroat trout were stocked into the upper Snake, Southeast, and Magic Valley regions of our study area. Records indicate that stocking ranged from a few dozen fish for one or two events to several million fish over multiple years (S2 Table). The spatial extent of translocations varied widely across the watershed at 278 locations (Fig 9). The South Fork of the Snake River and surrounding areas appear to have received the greatest intensity, whereas the Raft River and Portneuf River watersheds appear to have had much lower levels of stocking of cutthroat trout (Fig 9). Of the 30 populations sampled for this study, only four of the streams had records of fish introduced into those named streams. According to the records, Pine Creek, in the South Fork of the Snake River, had the most cutthroat trout translocated of all the populations sampled. There were no records of translocations for the sampled streams in the Raft River watershed; only high mountain lakes were reported to have experienced cutthroat trout stocking (Fig 9). In the Portneuf River, three populations had one or two stocking events (Gibson Jack, Pebble, and Toponce creeks) with most events occurring in mainstem rivers or reservoirs. Average nearest neighbor distance between cutthroat trout streams and reported stocking location was 44.72 km, with a range of 0 to 121.0 km.

**Fig 10:**
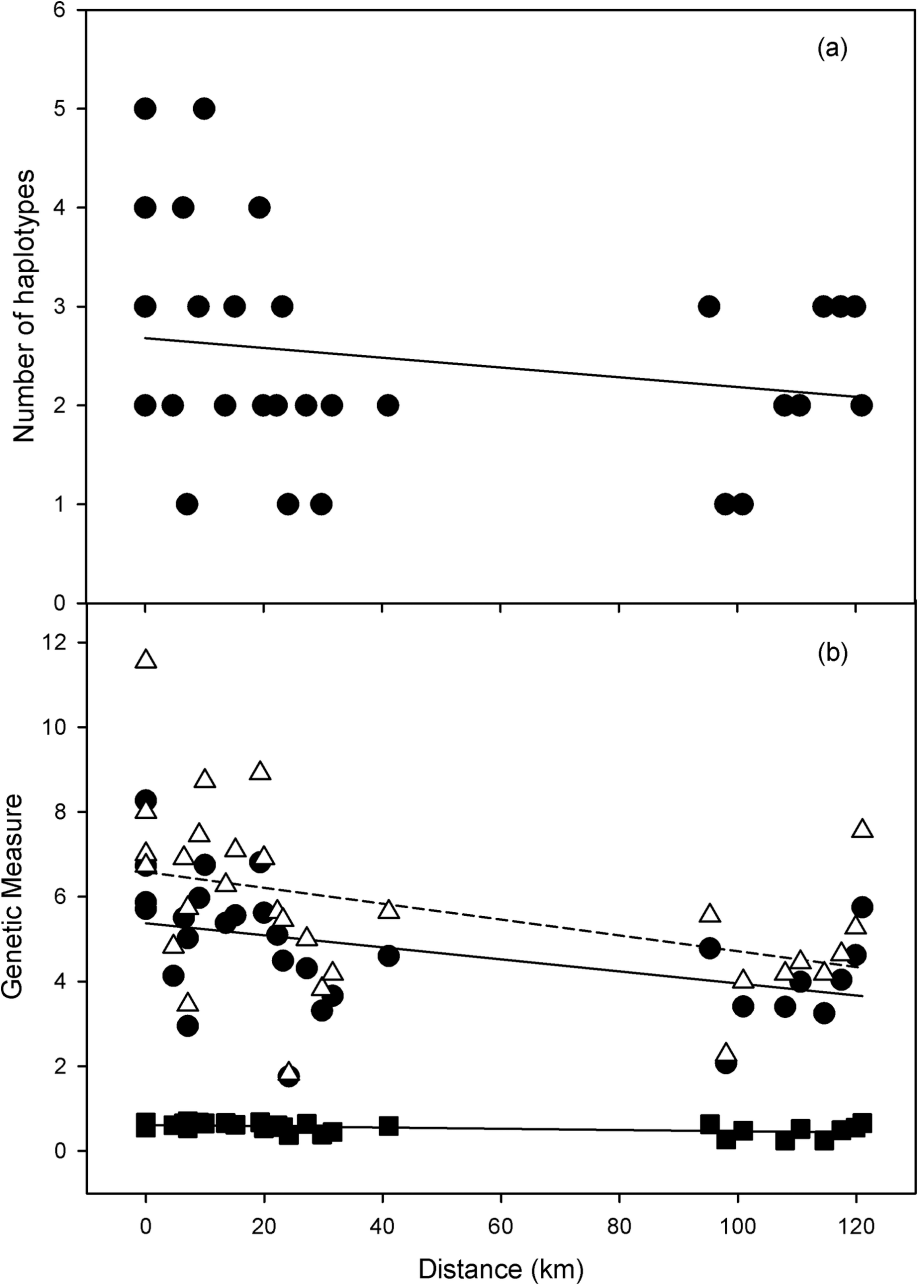
Distance to nearest stocking location versus genetic diversity. (a) The relationship between nearest stocking location distance (km) and number mtDNA haplotypes detected at a sampling location. (b)The relationship between nearest stocking location distance (km) and three genetic measures based on populations of cutthroat trout within the Bonneville Basin and upper Snake River. Solid circles and solid line represent average allelic richness (r^2^ = 0.196, n = 30, p = 0.014), open triangles and dotted line represent the average number of alleles (r^2^ = 0.171, n = 30, p = 0.023), and solid squares and dashed line represent average heterozygosity (r^2^ = 0.266, n = 30, p = 0.0035).

Spatial analyses of stocking data and genetic data indicated significant associations between some variables or a combination of variables and not others. Simple correlational analysis of the number of mtDNA haplotypes detected at a sampling location was weakly and negatively related to distance to the nearest stocking location (Fig 10A; r = -0.35, P = 0.059), and not significantly related to the number of fish stocked at the nearest location (r = 0.15, P = 0.44) or the number of stocking events at the nearest stocking location (r = 0.17, P = 0.37). However, when we combined all factors in a multiple regression analysis, the number of haplotypes detected was negatively correlated with distance to nearest stocking location (partial r = -0.35, F_1, 26_ = 5.10, P = 0.033), positively correlated to the number of stocking events (partial r = -0.42, F_1, 26_ = 7.30, P = 0.012), and negatively related to the number of fish stocked (partial r = -0.29, F_1, 26_ = 3.62, P = 0.068).

Microsatellite data also revealed similar associations of genetic diversity with measures of stocking history. Allelic richness was negatively correlated with distance to nearest stocking location (Fig 10B; r = -0.44, P = 0.014), but not significantly related to the number of fish stocked at the nearest location (r = 0.31, P = 0.091) or the number of stocking events at the nearest stocking location (r = -0.05, P = 0.79). Similarly, the number of alleles detected at microsatellite loci was negatively correlated with distance to nearest stocking location (Fig 10B; r = -0.41, P = 0.023), and was positively correlated with the number of fish stocked at the nearest stocking location (r = 0.36, P = 0.051), but not significantly related to the number of stocking events at the nearest location (r = -0.02, P = 0.92). Heterozygosity was also negatively correlated with distance to nearest stocking location (Fig 10B; r = -0.52, P = 0.0035), and not significantly correlated with number of stocking events (r = -0.23, P = 0.23) or total number of fish stocked (r = 0.23, P = 0.22) at the nearest stocking location. When all three stocking variables were included in separate multiple regression analyses of microsatellite data, similar patterns were observed. Allelic richness was significantly related to distance to nearest stocking location (partial r = -0.34, F_1, 26_ = 15.65, P = 0.0005) as was the number of stocking events (partial r = 0.094, F = 4.29, P = 0.048), but not the number of fish stocked (partial r < 0.01, F_1, 26_ < 0.01, P = 0.99). The number of alleles detected at a sampling location was negatively related to distance to nearest stocking location in multiple regression analysis (partial r = -0.31, F_1, 26_ = 14.38, P = 0.00080), positively related to the number of stocking events (partial r = 0.14, F_1, 26_ = 6.41, P = 0.018), and not related to the number of fish stocked analysis (partial r < 0.01, F_1, 26_ < 0.01, P = 0.97). Heterozygosity was negatively related to distance to nearest stocking location (partial r = -0.23, F_1, 26_ = 8.13, P = 0.00084), and not significantly related to the number of stocking events (partial r = -0.0001, F_1, 26_ = 0.04, P = 0.86), or the number of fish stocked (partial r = 0.032, F_1, 26_ = 1.13, P = 0.30).

## Discussion

In this study, we investigated whether the distribution of cutthroat trout in the Snake-Bonneville contact zone is explained by natural pathways of dispersal or by stocking of nonnative populations. Results of this, and previous studies, indicate that historical geographic features have played a significant role in the formation and organization of cutthroat trout diversity [41,81]. Interestingly, our analyses also suggest that stocking of cutthroat trout had minimal influence on natural distribution patterns, despite the intensity of stocking. Analyses of genetic diversity point to natural dispersal of two major lineages and three clades of cutthroat trout between the Bonneville Basin and upper Snake River watershed, providing evidence in support of paleodrainage connections. Such connections have been proposed by geologists and are of continued interest to biologists as an explanation for current distributions of fish fauna across the landscape [12,36,82,83].

As a slowly evolving molecule of the genome, mtDNA is often used to estimate deep evolutionary divergence between and within taxa [84,85]. For aquatic taxa, mtDNA has been particularly useful for uncovering the occurrence of movement barriers that naturally isolate watersheds over long periods of time. When geographic isolation is sustained, the distribution of mtDNA haplotypes can be used to identify historical barriers and connections [86,87]; however, secondary contact between lineages [88,89] and human-mediated translocation of taxa can obscure their natural extent, limiting the application of mtDNA data alone [90–92]. Across cutthroat trout populations, significant evolutionary divergence is reflected in distinct lineages that can be defined by mtDNA haplotypes. Coastal, westslope, Lahontan, and Rio Grande cutthroat trout, all have mtDNA haplotypes that appear to diagnose specific geographic areas and can therefore be used to define subspecific boundaries [41,93]. In other subspecies, intermixed mtDNA haplotypes may be the result of natural admixture from historical events or from more recent translocations [33,46,94]. While it would be logical to conclude that haplotypes from geographically distant locations are from non-native introductions, if admixed populations are in adjacent watersheds then one must be cautious when inferring whether the population is introduced or not [95]. In the current study, the distribution of mtDNA lineages in the contact zone was intermixed in some populations, but tended to decrease in frequency from locations downstream of possible paleodrainage connections (Fig 3). A downstream progression and intermixing of haplotypes from hypothesized points of connection can indicate secondary contact of evolutionary lineages by natural processes [95,96]. Given the admixed pattern, however, translocations are not an impossibility based on these results alone. While mtDNA can be used to infer evolutionary divergence, the examination of contemporary distribution patterns and connectedness also requires more polymorphic genetic markers.

Nuclear microsatellite loci are highly polymorphic and putatively selectively neutral, and thus are particularly useful for examining current geographic genetic structure among populations. In highly vagile species, microsatellite data reveals panmixia with little population structure except at very large spatial scales [97]. In contrast, dispersal-limited species exhibit population structure and increased genetic differentiation between neighboring populations [98]. Contact zones, like that between subspecies of cutthroat trout, pose a unique situation when trying to determine whether the populations in these ranges overlap as a consequence of historical connections or by recent introductions. Codominant microsatellite data can provide information about current population structure and is complimentary to the slower evolving, deeper divergence estimates provided by mtDNA data. While studies of cutthroat trout have investigated contemporary distribution patterns with nuclear data [93,99,100], secondary contact has not fully been explored with all subspecies that exhibit an intermixed distribution. In this study, microsatellite analyses indicate contemporary gene flow is restricted within watershed boundaries. These data, in conjunction with mtDNA analyses, support the explanation that historical connections provided natural avenues for dispersal between the Bonneville Basin and Snake River watershed and that current watershed boundaries have continued to limit gene flow since that dispersal event.

Extensive stocking of nonnative fishes has occurred in ecosystems worldwide and can have significant effects on native biodiversity through competition, predation and disease transmission [101–103]. When introduced populations have a close phylogenetic relationship with native taxa, hybridization and introgression can further complicate how to assess their status. Cutthroat trout are one of the most widely distributed freshwater fish in western North America, but they exhibit significant evolutionary diversification organized by geographic barriers not always understood or recognized [41,104]. Hatchery propagation of cutthroat trout from a handful of sources and widespread stocking of fish has raised concerns that conservation populations may not represent native populations [6,42,46]. Given the admixed distribution of haplotypes and the extensive stocking history of cutthroat trout, one possible explanation for the biogeographic pattern observed in the study area is from hatchery introductions. Taken together, however, mtDNA and microsatellite data indicate an organized genetic population structure with little influence of translocated fish. With records of more than 120 million cutthroat trout stocked into the study area, survival and success of those fish must have been extremely poor. In fact, hatchery propagated fish are well-known for their low survival rates when released into natural ecosystems [105–107]. For example, many millions of hatchery produced Pacific salmon and steelhead trout are released into the Columbia River every year to supplement natural populations, but often have much lower survival rates than their wild counterparts [108]. Hatchery populations of resident trout and char species also seem to have similarly low survival rates when released into lakes and streams [109–112]. Further evidence of low translocation success is reflected in the observation that no rainbow trout haplotypes were detected in our samples despite over 166 million rainbow trout being stocked in the study area between 1966 and 2016 (https://idfg.idaho.gov/fish/stocking). In the populations we sampled, most were far from stocking locations in headwater streams and typically isolated by movement barriers, making it difficult for hatchery fish to interact with these native populations.

The correlation between genetic diversity and the proximity to stocking was not unexpected. The introduction of individuals into a population should add genetic variation [113,114] and has been used to supplement small populations at risk of genetic loss. However, translocation of individuals outside their native range, even when supplementing threatened populations, must consider local adaptations and geographic structure, or how such actions can be detrimental [101,115,116]. In our study, any translocation effect was insufficient to disrupt the population genetic structure of cutthroat trout in the contact zone, indicating the natural organization is largely intact despite widespread stocking. Previous analyses of stocking history in salmonids also indicate that proximity of stocking locations influences whether non-native populations become introgressed with native populations [117,118].

Differentiation and geographically organized genetic population structure appears common among non-anadromous trout populations and is especially prominent among interior cutthroat trout subspecies. Past studies of resident trout populations have revealed similar findings to our results, with strong genetic differentiation hierarchically organized within and between watersheds and less differentiation at smaller spatial scales [93,100,119,120]. Salmonid fishes are commonly known to home to headwater streams for spawning and subsequent rearing of juveniles [121]. Cutthroat trout species are probably restricted to smaller streams for spawning and rearing, in part, because larger rivers may exhibit flow regimes and dynamics outside the range of suitable habitat for smaller-bodied trout species. As a result, strong selective pressure to return to natal streams may continually re-inforce the pattern of a highly organized genetic population structure found among cutthroat trout populations.

The exchange of aquatic taxa between neighboring watersheds illustrates how the gain and loss of natural connections over time can create species distribution patterns that do not adhere to watershed boundaries. In particular, changes in climate and hydrological conditions affect the extent and degree of connectivity between populations of aquatic organisms [11,16]. Because they are restricted within the landscape, fish are reliant on aquatic corridors to disperse and populations can become genetically distinct when natural connections are altered [122]. Cutthroat trout populations in the Snake-Bonneville range have experienced a long history of fluctuating watershed connections that appear to have influenced their distribution and population structure [33,41,81]. Pluvial Lake Bonneville began to rise with the addition of the Bear River to Lake Bonneville around 50 ± 10 ka [123]. At about 17,400 years ago, Lake Bonneville overflowed and the flood passed through Marsh Valley and the Portneuf Valley before entering the Snake River Plain [45]. This flood created a temporary watershed connection from the Bonneville Basin to the Portneuf River watershed, facilitating fish dispersal northward with the flow of water. The Bonneville flood provides an explanation for the current distribution of the three clades of cutthroat trout in the Portneuf River. While the combination of lineages in the Raft River could also be a result of the Bonneville flood, other studies have hypothesized that headwater transfer between the Raft River and rivers flowing southward into ancient Lake Bonneville is another possible avenue of dispersal [33,41]. Stream capture between the Raft River and streams flowing into Lake Bonneville may be another paleodrainage connection that explains the contemporary distribution patterns of cutthroat trout in the Snake-Bonneville contact zone.

Cutthroat trout are a species of conservation concern and efforts to improve their status have focused on removing non-native competitors or predators, restoring habitat, and by reintroduction programs [124–127]. As the distribution of cutthroat trout subspecies are largely defined by watershed boundaries [39], management decisions for a given subspecies are often applied using those boundaries, despite the possibility of secondary contact in transition zones. When a subspecies occurs in a neighboring watershed, stocking practices are often invoked as the explanation. This study illustrates the importance of understanding the evolutionary history of cutthroat trout subspecies, in conjunction with contemporary gene flow. Consistent with other studies, our study suggests that conservation decisions should consider the genetic structure between watersheds, as well as in neighboring populations [128]. While mtDNA haplotypes and genetic population structure may not align with all levels of intraspecific variation, they do describe primary axes of diversity that should inform how management plans proceed. To restore native populations, reintroduction efforts must consider localized adaptations, evolutionary lineages, secondary contact and differences between neighboring populations. By combining historical and contemporary genetic data, biologists are likely to provide the most comprehensive information to aid in conservation efforts.

### Conclusions

Natural pathways of dispersal appear to be a significant factor influencing the distribution of cutthroat trout in the Snake-Bonneville contact zone. Cutthroat trout populations have diversified into three phylogenetic clades intermixed in the Snake River and adjacent Bonneville Basin. Historical events appear to have shaped the distribution of these evolutionary lineages through geographic connections and isolation. However, human-mediated translocations of cutthroat trout into neighboring populations may have also influenced the distribution of genetic diversity by facilitating gene flow near stocking locations. Mitochondrial DNA data support historical aquatic connections that allowed dispersal of cutthroat trout into the upper Snake River watershed through the Bonneville Flood and headwater transfer with ancient Lake Bonneville. Microsatellite evidence identifies contemporary gene flow and migration that is primarily within watersheds and influenced by stream distance. While extensive stocking has occurred within the watersheds, these events appear to have had minimal influence on the natural distribution of populations sampled. Our study illustrates how genetic data can be used to identify native or introduced populations in a contact zone. Importantly, this information will help identify where historical connections may have existed and allow mangers to prioritize populations of conservation concern.

## Supporting information

S1 Table Pairwise F_ST_ (below diagonal) and geographic distance (km) (above diagonal) for cutthroat trout populations within the Bonneville Basin and upper Snake River. Population ID’s are defined by Table 1 and listed in the same order of the first column.
(XLSX)

S2 Table Location of cutthroat trout stocking events in the upper Snake, Southeast, and Magic Valley regions. Stocking records of cutthroat trout obtained from the Idaho Department of Fish and Game historical stocking records database (https://idfg.idaho.gov/fish/stocking) for the years 1967 to 2016.
(CSV)

S1 Dataset Mitochondrial sequence data for 18 unique cutthroat trout ND2 haplotypes within the Bonneville basin and upper Snake River. Haplotype ID's are defined by Table 2.
(TXT)

S2 Dataset Microsatellite genotype data for cutthroat trout within the Bonneville basin and upper Snake River. Population ID's are defined by Table 1.
(XLSX)
